# Screening Selection of Hydrogen Evolution‐Inhibiting and Zincphilic Alloy Anode for Aqueous Zn Battery

**DOI:** 10.1002/advs.202307667

**Published:** 2024-01-18

**Authors:** Luyao Wang, Shaojie Zhou, Kai Yang, Weiwei Huang, Shigenobu Ogata, Lei Gao, Xiong Pu

**Affiliations:** ^1^ CAS Center for Excellence in Nanoscience Beijing Key Laboratory of Micro‐Nano Energy and Sensor Beijing Institute of Nanoenergy and Nanosystems Chinese Academy of Sciences Beijing 101400 China; ^2^ School of Nanoscience and Engineering University of Chinese Academy of Sciences Beijing 100049 China; ^3^ Beijing Advanced Innovation Center for Materials Genome Engineering Institute for Advanced Materials and Technology University of Science and Technology Beijing Beijing 100083 China; ^4^ Center on Nanoenergy Research School of Chemistry and Chemical Engineering School of Physical Science and Technology Guangxi University Nanning 530004 China; ^5^ Department of Mechanical Science and Bioengineering Osaka University Osaka 560–8531 Japan

**Keywords:** alloy anode, charge transfer, hydrogen evolution, local strain, Zn battery

## Abstract

The hydrogen evolution reaction (HER) and Zn dendrites growth are two entangled detrimental effects hindering the application of aqueous Zn batteries. The alloying strategy is studied to be a convenient avenue to stabilize Zn anodes, but there still lacks global understanding when selecting reliable alloy elements. Herein, it is proposed to evaluate the Zn alloying elements in a holistic way by considering their effects on HER, zincphilicity, price, and environmental‐friendliness. Screening selection sequence is established through the theoretical evaluation of 17 common alloying elements according to their effects on hydrogen evolution and Zn nucleation thermodynamics. Two alloy electrodes with opposite predicted effects are prepared for experimental demonstration, i.e., HER‐inhibiting Bi and HER‐exacerbating Ni. Impressively, the optimum ZnBi alloy anode exhibits one order of magnitude lower hydrogen evolution rate than that of the pure Zn, leading to an ultra‐long plating/stripping cycling life for more than 11 000 cycles at a high current density of 20 mA cm^−2^ and 81% capacity retention for 170 cycles in a Zn‐V_2_O_5_ pouch cell. The study not only proposes a holistic alloy selection principle for Zn anode but also identifies a practically effective alloy element.

## Graphical abstract

A holistic alloy selection principle of hydrogen evolution‐inhibiting and zincphilic alloy anode for Zn battery is proposed through the theoretical evaluation of 17 common alloying elements, which is evidenced experimentally by two representative alloying elements with opposite effects (Bi and Ni).

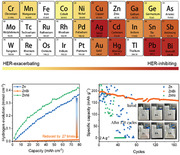

## 1 Introduction

The high cost and safety issues are still recognized as the major obstacles hindering the widespread applications of lithium‐ion batteries in grid energy storage, despite it is the most mature battery technology for electric vehicles.^[^ ^1^ ^]^ In this aspect, aqueous Zn‐ion batteries hold great promise to be the ultimate choice for stationary energy storage owing to their intrinsic high safety and low cost.^[^ ^2^ ^]^ Nevertheless, the thermodynamic instability of Zn metal anode in aqueous solution leads to parasitic side reactions, including spontaneous corrosion reaction and competitive hydrogen evolution reaction (HER) during the repeated Zn plating/stripping at the anode side.^[^ ^3^ ^]^ Meantime, the uncontrollable Zn dendrite growth further poses high risks of internal short circuit and cell failure.^[^ ^4^ ^]^ Studies have also shown that the HER can continuously interfere with Zn nucleation and deposition processes, thereby exacerbating the dendrite growth, and deteriorating the cell kinetics and reversibility.^[^ ^5^ ^]^ Therefore, the study of Zn metal anodes capable of suppressing HER and uncontrolled dendrite growth is of great significance for the development of practical ZIBs.

To date, various strategies for modifications of Zn anode have been investigated to tackle these issues aforementioned, such as Zn morphology designs, artificial interphases, and textured Zn deposition.^[^ ^6^ ^]^ Among them, Zn alloying strategy is a facile and effective approach. Recently, Zn‐X alloys (X = Ni, Sn, Mn, Cu, GaIn, Sb) and metal coatings (Cu, Ag, Au, Sn, In, Pb) have been investigated to overcome dendrite growth or hydrogen evolution problems.^[^ ^7^ ^]^ It is essential to introduce alloying metal elements that are zincophilic for Zn nucleation but resistive to hydrogen evolution. The introduction of zincophilic alloying elements by chemical displacement reaction, magnetron sputtering, rolling, or electrodeposition is beneficial for reducing the nucleation energy barrier of Zn, thereby significantly suppressing dendrite growth over repeated cycles.^[^ ^7^, ^8^ ^]^ Nevertheless, the effects on hydrogen evolution were not thoroughly studied for some reported alloying elements or metal coatings (Ni, Cu, Mn);^[^ ^7a,b,d^ ^]^ whereas, some elements were demonstrated to be effective in suppressing HER, such as Sn, In, Pb, Hg.^[^ ^7^, ^9^ ^]^ Furthermore, the cost and/or environmental friendliness of the alloying elements (Au, Ag, Pb, Hg, etc.) has not been well considered.^[^ ^7^, ^9^ ^]^ Therefore, global understanding and thorough evaluations are highly required for studying the effects of the doping elements on ZIB performances; meantime, generalized principles or criteria are urgently demanded to be established for screening selection of alloying dopants in Zn metal anodes.^[^ ^7^, ^10^ ^]^

In this work, we proposed to evaluate the alloying elements for Zn anode in a holistic way by considering their effects on HER, Zn nucleation, and environmental‐friendliness. Screening evaluation of 17 alloying elements was first conducted by theoretical calculation of their effects on hydrogen evolution and Zn nucleation thermodynamics. Their HER‐inhibiting capability and zincphilicity were sequenced. Based on the theoretical predictions, two alloying elements with opposite effects were selected for experimental demonstration, i.e., HER‐inhibiting Bi and HER‐exacerbating Ni. The on‐line hydrogen evolution measurements, repeated Zn plating/stripping tests, and Zn‐ion full‐cell performances all strongly supported the calculations. The optimum ZnBi alloy showed one order of magnitude lower hydrogen evolution rate than the pure Zn. Therefore, the ZnBi anode provided an ultra‐long plating/stripping cycling life for more than 11000 cycles at a high current density of 20 mA cm^−2^, and 81% capacity retention for 170 cycles in a Zn pouch cell without noticeable hydrogen evolution‐induced bulge.

## 2 Results and Discussion

### 2.1 Screening Selection of Alloying Elements Through Theoretical Calculations

**Figure 1:**
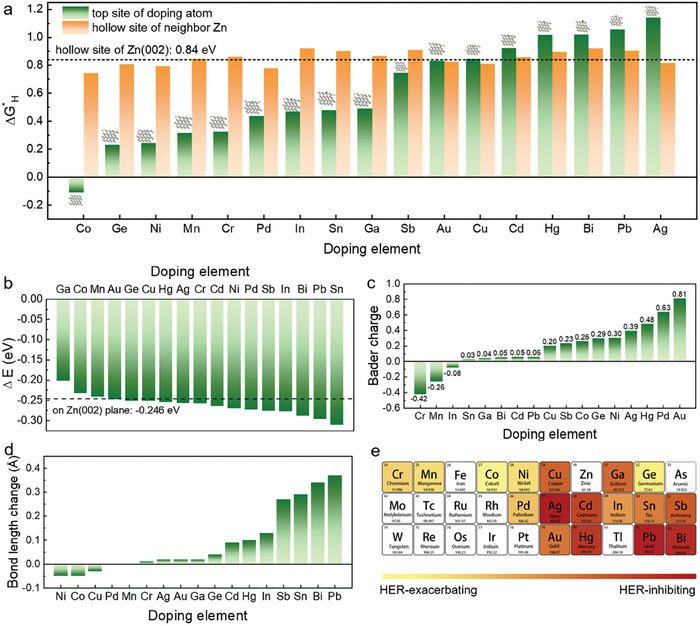
The criterion to select doping element to inhibit HER of Zn anode. a) The $\Delta G_{H}^{*}$ on the top site of the doping atom and the $\Delta G_{H}^{*}$ of the neighbor Zn hollow site. b) The binding energy of a Zn ad‐atom on the Zn (002) plane. c) The charge transfer between the doping atom and the surrounding Zn atom. d) The bonding length variation after doping. e) Periodic table of elements.

Alloying elements capable of inhibiting hydrogen evolution and promoting uniform Zn deposition are highly preferred for stable Zn metal anodes. Thermodynamically, it requires that the dopants should increase the energy barrier for the adsorption of H atoms but lower the energy barrier for the adsorption of Zn atoms on the Zn alloy surface. Considering the large number of possible doping elements, it is difficult to examine all potential alloying elements experimentally. Therefore, density‐functional theory (DFT) calculations are first employed to investigate the effects of alloying dopants on hydrogen evolution and Zn electrodeposition (**Figure** **1**). Seventeen possible metal elements were adopted for calculations (Figure 1e). First, their effects on HER are evaluated through the Gibbs free energy difference $\Delta G_{H}^{*}$ of H adsorption (Figure 1a), since it is regarded as a major parameter to evaluate the H_2_ evolution reaction activity.^[^ ^7^, ^10^ ^]^ High absolute value of $\Delta G_{H}^{*}$ can suggest excellent HER‐inhibiting capability.^[^ ^11^ ^]^ Detailed calculation methods are described in Note S1 (Supporting Information) and Figures S1–S3 (Supporting Information). The H adsorption on Zn(002) plane was examined, as Zn(002) is the closely‐packed plane with the minimum surface energy and can therefore be easier to be exposed. In our previous work, the (002) facets had the highest Δ*G*_H*_, which indicates the highest activation overpotential required for the HER.^[^ ^6h^ ^]^ Therefore, the $\Delta G_{H}^{*}$ on the pure Zn (002) plane was set as a reference value, i.e., 0.84 eV for a hollow site. Then, after substituting a Zn atom with an alloying metal atom, the $\Delta G_{H}^{*}$ of two sites were calculated, i.e., the top site of the doping atom and the hollow site of neighboring Zn matrix (Figure S2, Supporting Information). It is found that alloying dopants Cd, Hg, Bi and Pb can impressively not only exhibit high energy barrier at the top site of doping atom itself (0.920 eV, 1.017 eV, 1.020 eV, and 1.055 eV, respectively) but also raise the $\Delta G_{H}^{*}$ of the surrounding Zn matrix (0.855 eV, 0.896 eV, 0.919 eV, and 0.905 eV, respectively), suggesting excellent HER‐inhibiting capability. In contrast, elements such as Co, Ge, Ni, Mn, and Pd will exacerbate the hydrogen evolution, due to the significantly lowered $\Delta G_{H}^{*}$ at the sites on dopant top and/or in Zn matrix. Ag has the highest $\Delta G_{H}^{*}$ at top site among these elements (1.138 eV), but it lowers the $\Delta G_{H}^{*}$ of surrounding Zn matrix (0.815 eV); on the contrary, In, Sn, Cr, Ga and Sb are elements that have relatively low $\Delta G_{H}^{*}$ at the top site of itself but can increase the $\Delta G_{H}^{*}$ of surrounding Zn matrix (0.918 eV, 0.901 eV, 0.858 eV, 0.865 eV, and 0.908 eV, respectively). Au and Cu dopants seem to show trivial effect (only slightly decreased $\Delta G_{H}^{*}$) on the hydrogen evolution comparing with the pure Zn matrix.

Additionally, the binding energy of a Zn ad‐atom on the Zn(002) plane with an alloying atom was studied (Figure 1b). Comparing to the −0.246 eV of the pure Zn, only Co, Mn and Ga reduce the binding energy. The other fourteen alloying elements all show increased binding energy of the Zn ad‐atom, among which Bi, Pb and Sn show impressively improved binding energy of −0.288 eV, −0.296 eV, and −0.31 eV, respectively. The improved adsorption of Zn ad‐atoms by alloying elements could suggest zincphilic sites for Zn nucleation during electrodeposition, which can lower the Zn plating overpotential and improve the uniformity.

The diverse effects of doping elements on hydrogen evolution and Zn deposition of alloy anodes can be understood by the different changes in electronic structure and local strain induced by the alloying atoms. As for the HER, the $\Delta G_{H}^{*}$ at the top site of doping atoms is consistent with the trend of the corresponding elemental metals, such like that Hg, Bi, Pb and Ag are well‐known resistive to HER while Co and Ni can catalyze HER.^[^ ^11^, ^12^ ^]^ To understand the caused variation in the neighboring Zn matrix, the Bader charge or charge transfer between the doping atom and Zn atom was calculated (Figure 1c). It is found that there are two types of electronic structures that help suppress HER for the $\Delta G_{H}^{*}$ of neighboring Zn: (i) the doped element have small charge transfer (Sn, Ga, Bi, Cd, Pb); (ii) charge transfers from the doped atom (Cr, Mn, In) to the surrounding Zn atoms. Both cases will weaken the hydrogen adsorption capacity of the hollow site of the surrounding Zn, and therefore increase the Gibbs energy barrier of hydrogen evolution. Furthermore, according to the calculated bond length change induced by the alloying element (Figure 1d), the elements with large bonding length variation (Sn, Bi, Pb, Sb) improve Zn ad‐atom adsorption. It can be inferred that large change in the bond length will cause lattice distortion around them, and these distorted regions become the adsorption and nucleation centers of Zn, therefore large local strain improved Zn ad‐atom adsorption and the possible uniform Zn plating. Whereas, a large change in bond length will produce compressive strain on the surrounding Zn (Cd, Hg, In, Sn, Bi, Pb), which will weaken the ability of hydrogen adsorption and increase the Gibbs energy barrier of hydrogen evolution.^[^ ^13^ ^]^

As mentioned above, the selection of alloying atom should consider holistically the HER‐inhibiting capability, uniform Zn deposition, environmental‐friendliness and low cost. Fortunately, Bi could be a very satisfactory candidate. In, and Sn are two possible moderate choices with positive effects, as also been demonstrated experimentally.^[^ ^7c,i^ ^]^ Cu, as an alloying element, has trivial effect, but, as a metal substrate, can initiate hetero‐epitaxial growth of (002)‐textured Zn.^[^ ^6h^ ^]^ Pb, Hg, and Cd should be less considered for their toxicity, despite their impressive effects calculated herein and demonstrated experimentally in literature.^[^ ^7^, ^9^ ^]^ Au and Ag are expensive but can show positive effects, as also demonstrated by an experimental Zn‐Ag alloy anode.^[^ ^7e^ ^]^ Detrimental elements, such as Co, Ge, and Ni, should be definitely avoided. Special attentions should also be paid to excluding these impurities when manufacturing Zn anodes.

### 2.2 Fabrication and Characterization of Zn‐Bi and Zn‐Ni Alloy Anodes

**Figure 2:**
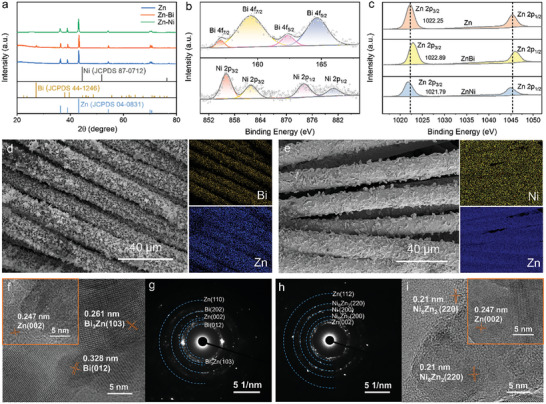
Characterization of alloy electrodes. a) XRD patterns of pure Zn, ZnBi, and ZnNi samples. b) High‐resolution XPS Bi *4f* and Ni *2p* spectra of ZnBi and ZnNi samples. c) XPS high‐resolution Zn *2p* spectra of pure Zn, ZnBi, and ZnNi samples. SEM and EDS mapping of d) ZnBi and e) ZnNi samples. HRTEM images f) ZnBi and i) ZnNi samples. SAED images g) ZnBi and h) ZnNi samples.

Above calculations and discussions have provided clear criteria for alloying design of Zn anode. Whereas, it is hard to fabricate all these different Zn alloys, so two representative alloy elements, i.e., Bi and Ni, are selected to demonstrate the viability of above predictions. Bi represents the both zincphilic and HER‐inhibiting alloying element, while the Ni is predicted to be HER‐exacerbating and potentially detrimental to Zn anode. We first fabricated the Zn‐Bi and Zn‐Ni alloy electrodes with various amounts of doping contents on 3D carbon cloth (CC) substrates via a simple electrodeposition method. The experimental part introduces the electrodeposition parameters of five samples (i.e., pure Zn, ZnBi, ZnBi‐2, ZnNi, and ZnNi‐2) in detail. We used inductively coupled plasma optical emission spectrometry to determine the content of elements in five samples. The atomic percentages of Bi in ZnBi and ZnBi‐2 anodes were 1.264 at.% and 4.513 at.%, respectively (Table S1, Supporting Information); the atomic percentages of Ni in ZnNi and ZnNi‐2 anodes were 1.376 at.% and 14.879 at.%, respectively (Table S2, Supporting Information). The X‐ray diffraction (XRD) pattern confirms the successful fabrication of alloying anodes. Besides the peaks of metallic Zn (JCPDS 04–0831), sharp characteristic peaks of metallic Bi (JCPDS 44–1246) was observed for ZnBi sample (**Figure** **2**a). The elemental Ni was not observed in XRD of ZnNi sample, but elemental Ni can be observed from the selected‐area electron diffraction (SAED) in Figure 2h, suggesting there could be possibly very small amount of element Ni.

The existence of the doping elements in the two samples (ZnBi and ZnNi) were further confirmed by X‐ray photoelectron spectroscopy (XPS) (Figure S4, Supporting Information). As shown in Figure 2b, the high‐resolution Bi 4f spectra of the ZnBi anode show two strong peaks at 162.4 and 157.1 eV, which can be assigned to Bi 4f_5/2_ and Bi 4f_7/2_ of zero‐valence Bi. The remaining peaks are at 164.6 eV and 159.5 eV, corresponding to Bi 4f_5/2_ and Bi 4f_7/2_ of Bi‐O species.^[^ ^14^ ^]^ The peaks at 880.9 eV and 861.75 eV correspond to Ni 2p_1/2_ and Ni 2p_3/2_ of Ni‐O species, respectively.^[^ ^15^ ^]^ These results indicate that the oxidation of the alloy anode surface is inevitable. Additionally, the Zn 2p XPS spectra of the bare Zn anode deliver two typical peaks of Zn 2p_3/2_ (1022.3 eV) and Zn 2p_1/2_ (1045.4 eV) (Figure 2c). Compared to bare Zn, the binding energy of Zn 2p_3/2_ in ZnBi is 0.6 eV higher, which is attributed to the formation of ZnBi alloy.^[^ ^16^ ^]^ In contrast, the characteristic peak of Zn 2p_3/2_ in the ZnNi sample shift to the lower binding energy. This downshift of XPS peak in ZnNi sample could be caused by the transfer of valence electron from the Ni atoms to the Zn atoms, which is consistent with literature.^[^ ^17^ ^]^ Therefore, the above results confirmed the successful deposition of Bi‐doped and Ni‐doped metal electrodes on the CC surface.

Scanning electron microscopy (SEM) was used to observe the surface morphology of different electrodes. The fiber diameter increased after electrodeposition, suggesting the successful loading of pure Zn, ZnBi, ZnBi‐2, ZnNi, or ZnNi‐2 alloy coatings in a relatively uniform manner (Figure 2d,e; Figure S5, Supporting Information). For the pure Zn sample, Zn particles with sharp surfaces were packed together (inset in Figure S5b, Supporting Information); whereas, the Zn‐Bi and Zn‐Ni alloy particles shows grainy morphology without exposed sharp crystal planes (inset in Figure S5c–f, Supporting Information). The homogenous distribution of Bi or Ni in the alloying samples was also confirmed by the energy dispersive spectroscopy (EDS) mapping (Figure 2d,e). Transmission electron microscopy (TEM) images of the ZnBi and ZnNi anodes show the grainy Zn alloy particles in hundreds of nanometer size and abundant sub‐10 nm inclusions uniformly distributed in the particles (Figure S6, Supporting Information). Here, inclusions of Bi_3_Zn and Ni_8_Zn_2_ were identified through high‐resolution transmission electron microscopy (HRTEM) images (Figure 2f,i) and selected‐area electron diffraction (SAED) patterns (Figure 2g,h) for ZnBi alloy and ZnNi alloy, respectively. The uneven local mass transport during the electrodeposition process may be a reason for the precipitates formation. Some precipitates not consistent with the thermal equilibrium phase diagram could even be possibly formed. These inclusions have also been observed in several recent studies.^[^ ^7^, ^10^, ^18^ ^]^

### 2.3 The Effect on Hydrogen Evolution by the Alloying Anodes

**Figure 3:**
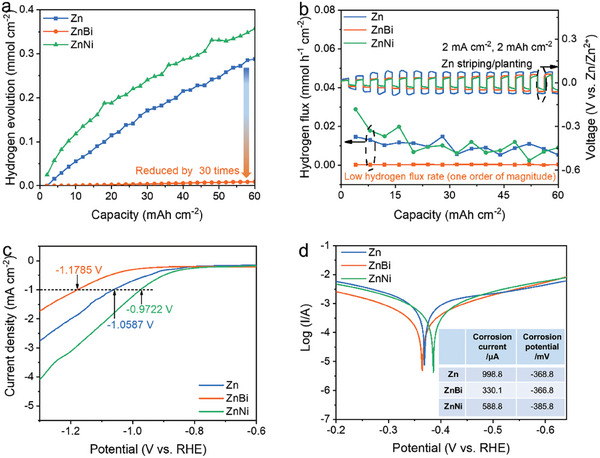
In situ measurement of a) hydrogen evolution and b) hydrogen evolution flux varying with the cumulative plating/stripping capacity of pure Zn, ZnBi, and ZnNi symmetric cells. c) Hydrogen evolution polarization curves of pure Zn, ZnBi, and ZnNi samples. d) Tafel curves of Zn, ZnBi, and ZnNi samples.

To demonstrate the ability of the alloying anode in suppressing or exacerbating the hydrogen evolution side reaction, we performed in‐situ measurements of hydrogen generation during Zn plating/stripping in symmetric cells. Specifically, we connected the all‐glass automatic gas analysis system with a gas chromatograph to realize on‐site monitoring of the hydrogen flux. In the symmetric cell test, the cut‐off capacity was set at 2 mAh cm^−2,^ and the current density was 2 mA cm^−2^. The hydrogen production (normalized by the electrode area) of different electrodes with the change of cumulative plating/stripping capacity was compared, and its influence on hydrogen production was evaluated (**Figure** **3**a,b). The hydrogen evolution of the ZnBi and ZnBi‐2 anodes is significantly less than that of the pure Zn electrode, while the hydrogen evolution of the ZnNi and ZnNi‐2 electrodes is higher than that of the pure Zn electrode (Figure 3a; Figure S7, Supporting Information). After 60 mAh cm^−2^ of plating/stripping cycling, the amount of hydrogen evolution of the ZnBi cell (0.009 mmol cm^−2^) was reduced by about 30 times compared with that of the pure Zn cell (0.288 mmol cm^−2^); on the contrary, the ZnNi cell (0.357 mmol cm^−2^) produced even higher amount of hydrogen than the pure Zn cell (Figure 3a). The ZnBi symmetric cell also showed extremely low hydrogen flux rate during the Zn plating/stripping process (about 0.000358 mmol h^−1^ cm^−2^), which is more than one order of magnitude than that of pure Zn cell (0.00541 mmol h^−1^ cm^−2^) and ZnNi cell (0.00908 mmol h^−1^ cm^−2^), respectively. We further obtained the hydrogen evolution polarization curves of the Zn, ZnBi, and ZnNi samples in 0.1 m Na_2_SO_4_ electrolyte solution by linear sweep voltammetry, which can show the onset potential of HER. As shown in Figure 3c, the HER potential shifted negatively from −1.0587 V (vs RHE) for Zn to −1.1785 V for ZnBi electrode and positively shifted to −0.9722 V for ZnNi electrode, suggesting that ZnBi alloy has higher HER overpotential while ZnNi has lower HER overpotential compared with pure Zn. Therefore, these experiment results are consistent with the above predicted calculations that Bi alloying is HER‐inhibiting but Ni alloying is HER‐exacerbating.

Furthermore, we analyzed the Tafel curves of the three samples (Figure 3d). Compared with Zn electrode (368.8 mV), the corrosion potentials of the ZnBi electrode (366.8 mV) positively shifted, but corrosion potentials of the ZnNi electrode is negatively shifted to 385.8 mV. The corrosion current of the ZnBi anode (330.1 µA) is only about one‐third of that of the pure zinc anode (998.8 µA), while the corrosion current of the ZnNi electrode maintains to be 588.8 µA. These results indicate that the corrosion reaction tends to be lower and the corrosion rate decreases due to the protective effect of Bi doping.

### 2.4 The Effect on Repeated Zn Plating/Stripping by the Alloying Anodes

**Figure 4:**
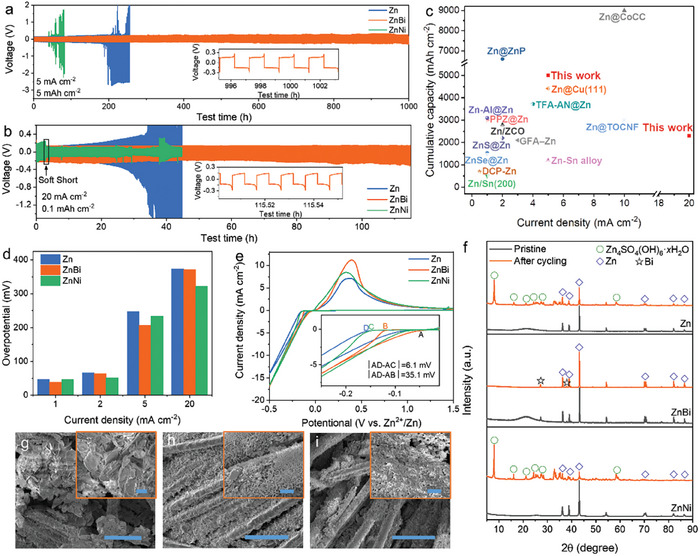
Comparison of the cycling performance of pure Zn, ZnBi, and ZnNi in symmetric cells at a) 5 mA cm^−2^ for 5 mAh cm^−2^ and b) 20 mA cm^−2^ for 0.1 mAh cm^−2^. c) Comparison of our designed work with literature on cumulative capacity recycled, cycle number, and current density. d) The overpotentials of the symmetric Zn, ZnBi, and ZnNi cells at the various current densities. e) The CV curves at a scan rate at 10 mV s^−1^ of the asymmetric Zn, ZnBi, and ZnNi cells. f) XRD pattern of Zn ZnBi and ZnNi electrodes before and after cycling. SEM images of g) pure Zn, h) ZnBi, and i) ZnNi samples after cycling. Scale bars, 50 µm (g–i) and 3 µm (insets of (g–i)).

The repeated Zn plating/stripping stability of Zn, ZnBi, ZnBi‐2, ZnNi, and ZnNi‐2 electrodes was then examined in symmetric cells. Comparing with 190 h cycling of the pure Zn cell at a current density of 5 mA cm^−2^ and cutting‐off capacity of 5 mAh cm^−2^, it is clearly observed that the ZnBi alloy electrode significantly elongated the cycling life to about 1000 h, while the ZnNi alloy electrode showed even much shorter cycling life of about 40 h (**Figure** **4**a). At a higher current density of 20 mA cm^−2^, similar trend was also obtained that the ZnBi alloy electrode achieved the longest cycling life for more than 115 h (Figure 4b). The voltage profile of the ZnNi suddenly decreases after 3 h, which is due to a “soft short circuit” under high current test conditions.^[^ ^19^ ^]^ The significantly improved performance of ZnBi alloy electrode while exacerbated performance of ZnNi alloy electrode was further confirmed at other testing current density conditions ranging from 1 mA cm^−2^ to 5 mA cm^−2^ (Figures S11 and S12, Supporting Information). We also compared our ZnBi alloy sample with several representative works in literature (Figure 4c; Table S3, Supporting Information).^[^ ^3^, ^6^, ^7^, ^20^ ^]^ The ZnBi sample exhibits excellent cumulative stripping/plating capabilities at higher current densities (Table S3, Supporting Information). It should be noted that the doping content also has a major effect on the performances (Figures S8–S10, Supporting Information). The ZnBi‐2 (4.513 at.%) and ZnNi‐2 (14.879 at.%) electrodes both showed worse Zn plating/stripping life than their counterparts with lower alloying contents. Therefore, ZnBi (1.264 at.%) and ZnNi (1.376 at.%) will be always used for following tests without specific notifications.

The dramatically improved Zn plating/stripping performances of ZnBi electrode can be attributed to the synergistic effects of the alloying elements on HER side reactions and Zn electrodeposition reactions. According to above theoretical calculations, the Bi and Ni could both lower the Zn nucleation overpotentials, which was consistent with the experiments (Figure 4d; Figure S13, Supporting Information). The overpotential of ZnBi and ZnNi cells maintained lower overpotential at different plating/stripping current densities. The Zn electrodeposition kinetics was further evaluated by cyclic voltammetry (CV) in Zn||Ti half cells. The nucleation process of metal ions had an intersection point (A), and the difference between the potential (B, C, or D) and the potential A is defined as the nucleation overpotential (Figure 4e). The nucleation overpotential of the ZnBi anode is 35.1 mV smaller than that of the pure Zn anode, which is bigger than the difference between of ZnNi anode and pure Zn anode. This result indicates that the overpotential can be reduced after Bi and Ni doping. Moreover, the higher current intensity of the CV curves indicates the more abundant active nucleation sites in the ZnBi alloy anode.^[^ ^21^ ^]^

Despite the comparably positive effects on Zn electrodeposition by both Bi and Ni alloying, their contrary effects on HER side reactions are the dominant reasons resulting in their opposite Zn plating/stripping performances. As shown by XRD tests in Figure 4f, after cycling for 40 hours at a current density of 2 mA cm^−2^ and cutoff capacity of 2 mAh cm^−2^, the ZnBi electrode surface still maintains the same metal characteristic peaks as the initial. In contrast, the Zn diffraction peaks on the surface of cycled pure Zn and ZnNi electrodes are obviously weaker, while the strong diffraction peaks of by‐products (Zn_4_SO_4_(OH)_6_·*x*H_2_O) are identified. These by‐products are associated with competing HER at the anode surface. Specifically, active water molecules compete with Zn^2+^ ions to be reduced into H_2_ during Zn deposition process, resulting in the consumption of H_2_O and ZnSO_4_, the increase of local pH value, and the production of solid non‐conductive by‐products Zn_4_SO_4_(OH)_6_·*x*H_2_O.^[^ ^5^, ^22^ ^]^ The Ni alloying could exacerbate the detrimental HER reaction, which therefore could lower the cell reversibility, deteriorate the cell impedance, and shorten the Zn plating/stripping life; whereas, the Bi alloying could suppress the HER reaction and achieve improved performances.

As also confirmed by the SEM morphology, the surface of the ZnBi electrode was still uniform and dense after plating/stripping at a current density of 2 mA cm^−2^ and cutoff capacity of 2 mAh cm^−2^ for 40 h (Figure 4h). The good reversibility and stability of the ZnBi electrode are mainly attributed to the fact that the ZnBi alloy can effectively induce the uniform deposition of Zn and suppress side reactions. However, obvious charging phenomena appeared when obtaining the SEM images of pure Zn and ZnNi anodes, which indicated that a large number of non‐conductive by‐products existed on the electrode surface (Figure 4g,i). The electrochemical impedance spectroscopy (EIS) in Figure S14 (Supporting Information) also supported above discussions. The initial charge transfer resistance (*R_ct_*) of the ZnBi symmetric cell (5.8 Ω) was lower than that of the pure Zn symmetrical cell (20.2 Ω) and ZnNi symmetrical cell (14.0 Ω). The violent side reactions of the pure Zn electrode and ZnNi electrode lead to a sharp increase of *R_ct_* after cycling (75.52 Ω and 57.12 Ω, respectively); whereas the ZnBi electrode showed only a very slight increase (13.97 Ω). The plating/stripping Coulombic efficiency (CE) tests of Zn||Cu asymmetric cells further confirmed the excellent reversibility and stability of ZnBi alloy electrodes. As shown in Figure S15 (Supporting Information), the ZnBi||Cu cell showed a stabler and higher average CE (99.2%), compared with the asymmetric bare Zn||Cu cell and ZnNi||Cu cell. Therefore, all above theoretical and experimental results are consistent.

### 2.5 The Full‐Cell Performances of the Alloying Anodes

**Figure 5:**
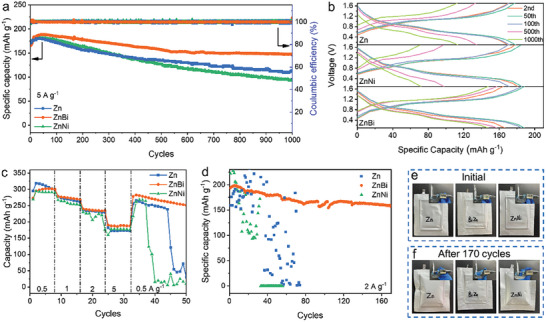
Performances of Zn‐V_2_O_5_ cells. a) Cycling performance of Zn||V_2_O_5_, ZnBi||V_2_O_5_ and ZnNi||V_2_O_5_ cells at 5 A g^−1^. b) The typical GCD curves of Zn||V_2_O_5_, ZnBi||V_2_O_5_ and ZnNi||V_2_O_5_ cells at the current density of 5 A g^−1^. c) Rate performances of Zn||V_2_O_5_, ZnBi||V_2_O_5_ and ZnNi||V_2_O_5_ cells. d) Cyclic stability of Zn||V_2_O_5_, ZnBi||V_2_O_5_ and ZnNi||V_2_O_5_ pouch cells. Digital photograph of Zn||V_2_O_5_, ZnBi||V_2_O_5_ and ZnNi||V_2_O_5_ cells e) before and f) after cycling.

The effectiveness and practicability of the alloy electrodes in full cells were evaluated with a V_2_O_5_ cathode. As shown in **Figure** **5**a, after 42 activation cycles, the ZnBi||V_2_O_5_ cell reached a maximum specific capacity of 188.8 mAh g^−1^ and then achieved a capacity retention rate of 88.59% after 1000 cycles at 5 A g^−1^ current density. In contrast, the capacity retention rates of ZnNi||V_2_O_5_ (93.6 mAh g^−1^) and Zn||V_2_O_5_ (113.7 mAh g^−1^) after the initial activation process are only 55.18% and 65.61%, respectively. Moreover, the average Coulombic efficiency of ZnBi||V_2_O_5_ cell is 99.99%, which is higher than that of ZnNi||V_2_O_5_ cell (99.89%) and Zn||V_2_O_5_ cell (99.92%). The corresponding charge/discharge voltage profiles are shown in Figure 5b, which also show the higher specific capacity and stable cyclability of the ZnBi||V_2_O_5_ cell than the Zn||V_2_O_5_ and ZnNi||V_2_O_5_ cell. As for the rate capability, despite the comparable initial capacities of the three cells at 0.5 A g^−1^, the ZnBi||V_2_O_5_ cell still shows slightly better rate performances than the other two cells (Figure 5c). As demonstrated above, these better performances in full cells can also be attributed to the HER‐inhibiting and Zn deposition‐promotion characteristics of ZnBi alloy electrode. The inhibited HER at anode side can suppress the formation of non‐conductive and irreversible byproducts, maintain the local pH environment, and therefore improve the reversibility and elongate the cycling stability of the full cell.

To verify the application potential of the alloy electrode and better show the effect of HER, we assembled Zn||V_2_O_5_ pouch cells without an air vent. The capacity of the ZnBi||V_2_O_5_ battery was only slightly worse than that of the pristine battery after 170 cycles (81% retention rate); whereas the capacity of the ZnNi||V_2_O_5_ and Zn||V_2_O_5_ battery fluctuated significantly and failed after only dozens of cycles (Figure 5d). The shape changes of the pouch cells strongly support the finding that the Bi alloy suppresses HER (Figure 5e,f). After 170 cycles at a current density of 2 A g^–1^, the thickness of the ZnNi||V_2_O_5_ and Zn||V_2_O_5_ pouch cells increased by 6.9 mm and 6.3 mm, respectively. The bulge of these two cells clearly showed the violent HER side reactions, which accounts for the capacity fluctuation and cell failure. However, the shape change of the ZnBi||V_2_O_5_ cell is limited after the same cycling process (Figure 5f). The ZnBi||V_2_O_5_ battery can also stably power the heart‐shaped lamp for 5 min (Figure S16, Supporting Information).

## 3 Conclusions

In conclusion, we established a refined screening index for the selection of dopant elements in alloyed Zn metal anodes through theoretical calculations of the HER and Zn nucleation thermodynamics. By taking into consideration of the price and environmental‐friendliness holistically, Bi was identified as a very promising alloying candidate with both high HER‐inhibiting capability and zincphilicity. To demonstrate the effectiveness of the screening sequence, Zn‐Bi and Zn‐Ni alloys were prepared for comparison studies with the pure Zn, where the Zn‐Ni alloy was selected as a control sample due to its predicted representative HER‐exacerbating effect. Experimental data of quantitative hydrogen evolution, repeated Zn plating/stripping in symmetric cells and cycling in Zn full cells were all consistent with the theoretical predictions. The optimum ZnBi anode can provide a long plating/stripping cycle life exceeding 115 h even at a high current density of 20 mA cm^−2^. This study not only proposed a holistic alloy selection sequence for Zn anode theoretically, but also demonstrated a practically effective Zn alloy anode. In future practical applications, smelting and rolling processing can be considered to better control the alloy content and type.

## Conflict of Interest

The authors declare no conflict of interest.

## Author Contributions

L.Y.W. and S.J.Z. contributed equally to this work. X.P., L.G., and L.Y.W. conceived the project and designed the experiments. L.Y.W. contributed to sample preparation and experiments. W.W.H. and S.J.Z. performed the theoretical calculation. L.Y.W., W.W.H., and S.J.Z. contributed to data analysis. All authors discussed the results and commented on the manuscript. L.Y.W., X.P., and L.G. wrote the paper with input from all authors.

## Supporting information

Supporting Information

## Data Availability

The data that support the findings of this study are available from the corresponding author upon reasonable request.
