# Supplementary material for: Screening Selection of Hydrogen Evolution‐Inhibiting and Zincphilic Alloy Anode for Aqueous Zn Battery
Source: Adv Sci (Weinh). 2024 Jan 18;11(12):2307667. doi: 10.1002/advs.202307667 (PMC10966540; doi:10.1002/advs.202307667)
Supplement: Supplementary file 1 — Supporting Information [file ADVS-11-2307667-s001.pdf]

ADVANCED SCIENCE
Open Access

Supporting Information

for *Adv. Sci.*, DOI 10.1002/advs.202307667

Screening Selection of Hydrogen Evolution-Inhibiting and Zincphilic Alloy Anode for Aqueous Zn Battery

Luyao Wang, Shaojie Zhou, Kai Yang, Weiwei Huang, Shigenobu Ogata, Lei Gao and Xiong Pu**

Supporting information

Screening selection of hydrogen evolution-inhibiting and zincphilic alloy anode for aqueous Zn battery

Luyao Wang[a,c], Shaojie Zhou[b], Kai Yang[a,e], Weiwei Huang[b], Shigenobu Ogata[d]，Lei Gao[b,d], Xiong Pu[a,c,e*]*

a CAS Center for Excellence in Nanoscience, Beijing Key Laboratory of Micro-Nano Energy and Sensor, Beijing Institute of Nanoenergy and Nanosystems, Chinese Academy of Sciences, Beijing 101400, China.

b Beijing Advanced Innovation Center for Materials Genome Engineering, Institute for Advanced Materials and Technology, University of Science and Technology Beijing, Beijing 100083, China.

c School of Nanoscience and Engineering, University of Chinese Academy of Sciences, Beijing, 100049, China.

d Department of Mechanical Science and Bioengineering, Osaka University, Osaka 560-8531, Japan.

e Center on Nanoenergy Research, School of Chemistry and Chemical Engineering, School of Physical Science and Technology, Guangxi University, Nanning 530004, China.

*corresponding authors. E-mail: puxiong@binn.cas.cn; gaolei@ustb.edu.cn

Experimental section

Pre-plating treatment of anodes: The anodes on hydrophilic carbon cloth were prepared by electrochemical deposition method using a two-electrode configuration. And the zinc sheet is used as the counter electrode. Firstly, the hydrophilic 0.33 mm thick carbon cloth (CeTech wos1009) was treated in 10 vol % HNO_3 (Beijing Chemical industry, Ar) solution under 0.05 A/cm^2 constant current process for 120 s and was washed with deionized water.

Fabrication of Zn anodes: The electrolyte used for the electrodeposition of the Zn electrode consists of 0.3 M $ZnSO_4 \cdot 7H_2O$ (Aladdin, Ar), 0.2 mM Sodium dodecyl sulfate (Macklin, Ar), 0.3 M $C_6H_5Na_3O_7.2H_2O$ (Aladdin, Ar), 0.04 M EDTA (Aladdin, Gr), 1 g/L polyethylene glycol (Mn = 2000) (Sigma-Aldrich). Then, the electroplating solutions were adjusted to pH 4.2~4.5 with 2 M NaOH (Aladdin, 97%) solution. Electrodeposition of anodes was carried out at constant current density j=0.025 A/cm^2 at 25 °C for 60 min using a three-electrode configuration. Subsequently, the anodes were washed several times with deionized water. The thickness of Zn sample after electrodeposition is 0.60 mm.

Fabrication of Zn-Bi alloy anodes: The electrolyte used for the co-electrodeposition of the Zn-Bi electrode consists of 0.3 M $ZnSO_4 \cdot 7H_2O$ (Aladdin, Ar), 0.2 mM Sodium dodecyl sulfate (Macklin, Ar), 0.3 M $C_6H_5Na_3O_7.2H_2O$ (Aladdin, Ar), 0.04 M EDTA (Aladdin, Gr), 1 g/L polyethylene glycol (Mn = 2000) (Sigma-Aldrich), 2 mM $Bi(NO_3)_3 \cdot 5H_2O$ or 10 mM $Bi(NO_3)_3 \cdot 5H_2O$ (Aladdin, Ar). Then, the electroplating solutions were adjusted to pH 4.2~4.5 with 2 M NaOH (Aladdin, 97%)

solution. Electrodeposition of anodes was carried out at constant current density j=0.025 A/cm^2 at 25 °C for 60 min using a three-electrode configuration. Subsequently, the anodes were washed several times with deionized water and were dried at 80 ℃ in a vacuum oven for 6 h. Finally, two samples were prepared: (1) ZnBi sample, using electrodeposition electrolyte containing 2 mM $Bi(NO_3)_3$; (2) ZnBi-2 sample, using electrodeposition electrolyte containing 10 mM $Bi(NO_3)_3$. After electrodeposition, the thickness of ZnBi and ZnBi-2 samples is 0.63 mm and 0.65 mm, respectively.

Fabrication of Zn-Ni alloy anodes: The electrolyte used for the co-electrodeposition of Zn-Ni electrodes consists of 0.3 M $ZnSO_4·7H_2O$ (Aladdin, Ar), 0.2 mM Sodium dodecyl sulfate (Macklin, Ar), 0.3 M $C_6H_5Na_3O_7.2H_2O$ (Aladdin, Ar), 0.04 M EDTA (Aladdin, Gr), 1 g/L polyethylene glycol (Mn = 2000) (Sigma-Aldrich), 5 mM $NiSO_4·6H_2O$ or 10 mM $NiSO_4·6H_2O$ (Macklin, Ar). Then, the electroplating solutions were adjusted to pH 4.2~4.5 with 2 M NaOH (Aladdin, 97%) solution. Electrodeposition of anodes was carried out at constant current density j=0.025 A/cm^2 at 25 °C for 60 min using a three-electrode configuration. Subsequently, the anodes were washed several times with deionized water and were dried at 80 ℃ in a vacuum oven for 6 h. Finally, two samples were prepared: (1) ZnNi sample, using an electrodeposition electrolyte containing 5 mM $NiSO_4$; (2) ZnNi-2 sample, using an electrodeposition electrolyte containing 10 mM $NiSO_4$. After electrodeposition, the thickness of ZnNi and ZnNi-2 samples is 0.46 mm and 0.45 mm, respectively.

Fabrication of MA-V_2O_5 cathode: The 1 g urea (Sigma-Aldrich, Ar), 0.75g NaI (Aladdin, 99%), and 0.9 g V_2O_5 (Sigma-Aldrich, Ar) were dissolved into deionized

water (50 mL). Then 500 uL H_2SO_4 (Beijing Chemical industry, Ar) was added to the solution and continuous stirring followed for 20 min. After that, the above solution was transferred to a 100 ml Teflon autoclave and treated for 3 h at 120 ^oC. After the autoclave was naturally cooled to room temperature, the product was filtered and washed with alcohol and deionized water. Finally, the green powder was dried at 80 °C for 24 h. The tested MA-V_2O_5 cathode was fabricated by mixing the MA-V_2O_5 (70 wt%), acetylene black (20 wt%) (maya, Ar), and polytetrafluoroethylene (10 wt%) (N-buliv) with some drops of anhydrous ethanol to form a slurry. The slurry was then rolled onto a stainless steel mesh and dried under vacuum at 80 ^oC for 2 h.

Materials characterization: The product morphologies were observed by scanning electron microscopy with an energy-dispersive X-ray spectroscopy (EDS) detector (SEM, Nova NanoSEM 450). The microstructures components of electrodes were observed by field emission scanning electron microscopy (FESEM, JEOL JEM-F200), selected-area electron diffraction (SAED), and high-resolution transmission electron microscopy (HRTEM, JED-2300T) at an acceleration voltage of 200 kV, respectively. The crystal structures and compositions of electrodes were investigated by X-ray diffraction (XRD, Ultima IV Cu K-Alpha radiation). The surface chemical compositions and states of electrodes were measured by X-ray photoelectron spectroscopy (XPS, Thermo Scientific K-Alpha). The atomic percentages of electrodes were measured by measuring Inductively Coupled Plasma Optical Emission Spectrum (ICP-OES, Agilent 725-OES).

Electrochemical measurements of symmetric and asymmetric cells: The

stripping/plating tests and coulombic efficiency of samples were performed in symmetrical cells using 2 M $ZnSO_4$ solution as the electrolyte on a battery test system (CT2001A, LAND, and CT-4008, Neware). The depth of discharge for Zn-Bi, Zn-Ni alloy and Zn electrode was 28.3%, 35.4% and 29.1% at 5 mA cm^{-2}, respectively. The cyclic voltammetry (CV) and electrochemical impedance spectroscopy (EIS) were measured by CHI 760E electrochemical workstation (CHI 760E, Chenhua, Shanghai) using 2 M $ZnSO_4$ solution as the electrolyte. The amount of electrolyte in standard CR2032 coin-type cells is 200 μl. Hydrogen evolution polarization curves and Tafel curves were measured by rotating electrode (Model 636A, Princeton) and CHI 760E electrochemical workstation (CHI 760E, Chenhua, Shanghai) in 0.1 M Na_2SO_4 solution as the electrolyte. The gas chromatographic (GC2002, Kechuang, Shanghai) and the all-glass automatic online trace gas analysis system were used to monitor the in-situ hydrogen evolution flux (Labsolar 6A, Perfectlight). Specifically, the symmetric battery test was carried out in a closed glass container. The gas produced in the container was sampled by the all-glass automatic online trace gas analysis system, and the amount of hydrogen in the gas was obtained by gas chromatography.

Electrochemical measurements of Zn||V_2O_5 full batteries: For the full battery, Zn||V_2O_5 full batteries were assembled in standard CR2032 coin-type cells using 2 M $Zn(CF_3SO_3)_2$ (Aladdin, Ar) solution as the electrolyte and Whatman glass fiber (GF/D) as the separator in air atmosphere. The amount of electrolyte in standard CR2032 coin-type cells is 200 μl. The typical GCD curves were collected on a Neware battery test system with a potential range of 0.1-1.7 V at room temperature. And we also evaluated

the effectiveness and practicability of the alloy anode in Zn-V_2O_5 pouch batteries. The amount of electrolyte in Zn-V_2O_5 pouch is 1500 μl.

Calculation methods: The DFT calculations were carried out using the Vienna Ab initio Simulation Package (VASP).[1] The core electrons were modeled with the projector-augmented-wave (PAW) method.[2] The exchange and correlation function of Perdew-Burke-Ernzerhof (PBE) was adopted within the framework of generalized gradient approximation (GGA).[3] The plane-wave basis cut-off energy was set to 400 eV. During the geometrical optimizations, the forces on all the relaxed atoms were less than 0.02 eV/Å.

The Zn(001) alloying supercells consisting of 4-layer atomic structures with sizes of 4×4×4 unit cells were adopted in this study. The K-points were set as 5×5×1 according to the Monkhorst-pack method. The alloying element was doped in the top layer of the Zn(001) supercell. For each supercell, the top site of the doping element and hollow site of neighbor Zn atoms were selected to investigate the effects of doping on hydrogen evolution performances.

The 4×4 slab models with X atoms doped on the surface of Zn(001) is constructed, and each model is arranged in the order of Cd, Cr, Pb, In, Pd, Hg, Ag, Bi, Co, Cu, Mn, Ni, Au, Ge, Sn, Ga and Sb atoms doping. The optimized surface doping structure model is shown in Figure S1 to calculate the energy of the system. In our previous work, it has been shown that the 4×4 ZnSn(001) slab system is the most effective in inhibiting hydrogen evolution.[4] In order to study the influence of X atoms on the surface hydrogen evolution behaviors, tow typical sites(X top and Zn hollow) were selected to

calculate the Gibbs free energy change ΔG_H*. Here, the two typical sites of ZnCd system were taken as an example as shown in Figure S2.

The Gibbs free energy changes ΔG_H^* of H adsorption on top site of doping element and hollow site of neighbor Zn were calculated according to the following equation:[5]

$$\Delta G = \Delta E_H + \Delta E_{ZPE} - T\Delta S_H$$

where ΔE_{ZPE} is the difference in zero point energy and ΔS_H is the entropy between hydrogen adsorption and hydrogen in the gas phase. Here the contributions from the electrode to both ΔE_{ZPE} and ΔS_H are small and could be neglected. As a result, the ΔE_{ZPE} and ΔS_H are obtained by,

$$\Delta E_{ZPE} = E_{ZPE}^{H} - 1/2E_{H^2}$$

$$\Delta S_H \cong -1/2S_{H^2}^{0}$$

and $S_{H^2}^{0}$ is the entropy of H_2 gas at the standard condition.

The binding energy of H atom adsorption on the surface of the supercell is obtained according to the following equation.

$$\Delta E_H = E_{sub}^{H} - E_{sub} - 1/2E_{H^2}$$

where E_{sub}^{H} is the energy of a H atom adsorbed on the electrode substrate, E_{sub} is the energy of the substrate and E_{H^2} is the energy of gas H_2.

Alloy doping also changes the dendrite phenomenon on the zinc electrode surface, which is reflected in its effect on the deposition process of Zn atoms on the Zn(001) surface. The slab model of Zn atom adsorption on ZnX(001) surface is constructed to find the reasonable adsorption position of Zn atom closest to doped at X atoms. The

structures of each system are shown in Figure S3.

In addition, the Bader charge transfer between the doping atom and the surrounding Zn atom was calculated to present the chemical interaction activity between the doping atom and Zn atom, and the bond length variation between the doping atom and the surrounding Zn atom was also investigated to demonstrate the local strain induced by the doping atom.

In Figure 5a, the loading level of the active material in the electrodes are Zn (1.989 mg/cm^2), ZnBi (2.336 mg/cm^2), ZnNi (2.595 mg/cm^2), respectively. The capacity balance between the cathode and anode (N/P ratio) in full cells are Zn (22), ZnBi (19), ZnNi (14), respectively. In Figure 5c, the loading level of the active material in the electrodes are Zn (2.568 mg/cm^2), ZnBi (2.711 mg/cm^2), ZnNi (1.775 mg/cm^2), respectively. The N/P ratio in full cells are Zn (17), ZnBi (17), ZnNi (20), respectively. In Figure 5d, the loading level of the active material in the electrodes are Zn (1.486 mg/cm^2), ZnBi (1.526 mg/cm^2), ZnNi (1.656 mg/cm^2) , respectively. The N/P ratio in full cells are Zn (23), ZnBi (23), ZnNi (17), respectively.

Table S1. Weight and atomic ratios of Zn and Bi in the Zn-Bi nanoparticle.

Sample	Zn	Bi
**ZnBi**	98.736	1.264
**ZnBi-2**	95.487	4.513

Table S2. Weight and atomic ratios of Zn and Ni in the Zn-Ni nanoparticle.

Sample	Zn	Ni
**ZnNi**	98.624	1.376
**ZnNi-2**	85.121	14.879

Table S3. Comparison of the Zn plating/stripping performances in symmetric cells between our Zn-Bi alloy electrode and other reported works

Electrode	Electrolyte	Current density (mA cm^{-2})	Capacity (mAh cm^{-2})	Life (h)	Cumulative capacity (mAh)	Cycle number	Ref.
ZnBi	2 M $ZnSO_4$	5	5	1000	5000	500	This work
		20	0.1	115	2300	11500	
		2	2	1600	3200	800	
Zn@ZnP	2 M $ZnSO_4$	2	0.5	3300	6600	6600	[6]
Zn/ZOC	2 M $ZnSO_4$	2	2	1400	2800	700	[7]
Zn/Sn(200)	1 M $ZnSO_4$	1	1	500	500	250	[8]
ZnS@Zn	1 M $ZnSO_4$	2	2	1100	2200	550	[9]
GFA-Zn	1 M $ZnSO_4$	3	3	700	2100	350	[10]
PPZ@Zn	2 M $ZnSO_4$	1	0.5	3000	3000	3000	[11]
ZnSe@Zn	2 M $ZnSO_4$	1	1	1530	1530	765	[12]
DCP-Zn	2 M $ZnSO_4$	0.5	0.1	1400	700	3500	[13]
TFA-AN@Zn	1 M $ZnSO_4$	4	2	930	3720	930	[14]
Zn-Al@Zn	2 M $ZnSO_4$	1	1	3100	3100	1550	[15]
Zn@Cu(111)	2 M $ZnSO_4$	5	1	880	4400	2200	[16]
Zn@TOCNF	2 M $ZnSO_4$	10	1	300	3000	1500	[17]
Zn-Sn alloy	2 M $ZnSO_4$	5	5	240	1200	120	[4]
Zn@CoCC	1 M $ZnSO_4$	10	1	900	9000	4500	[18]

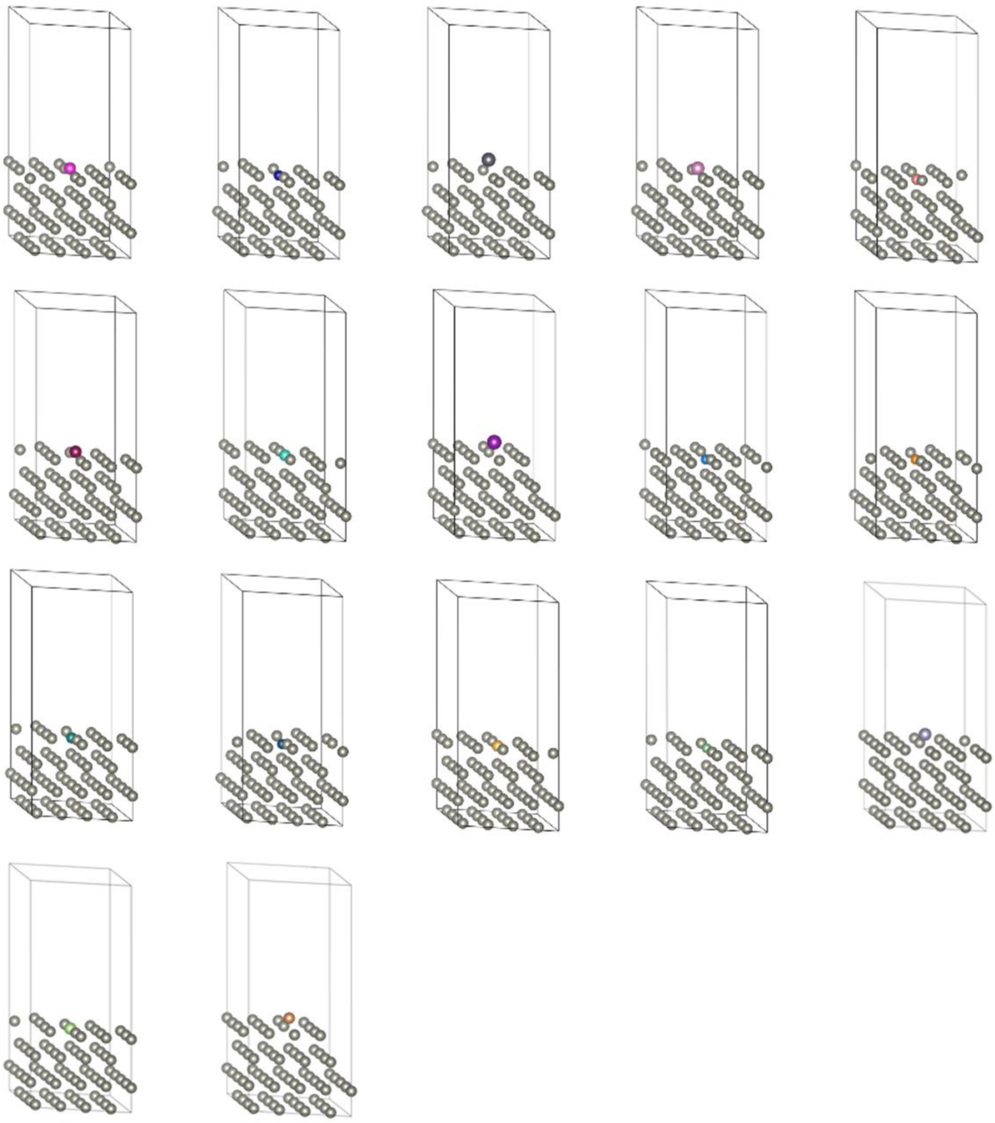

Figure S1. Zn(001) surface doped with X atoms (doped elements X in order of Cd, Cr, Pb, In, Pd, Hg, Ag, Bi, Co, Cu, Mn, Ni, Au, Ge, Sn, Ga, Sb)

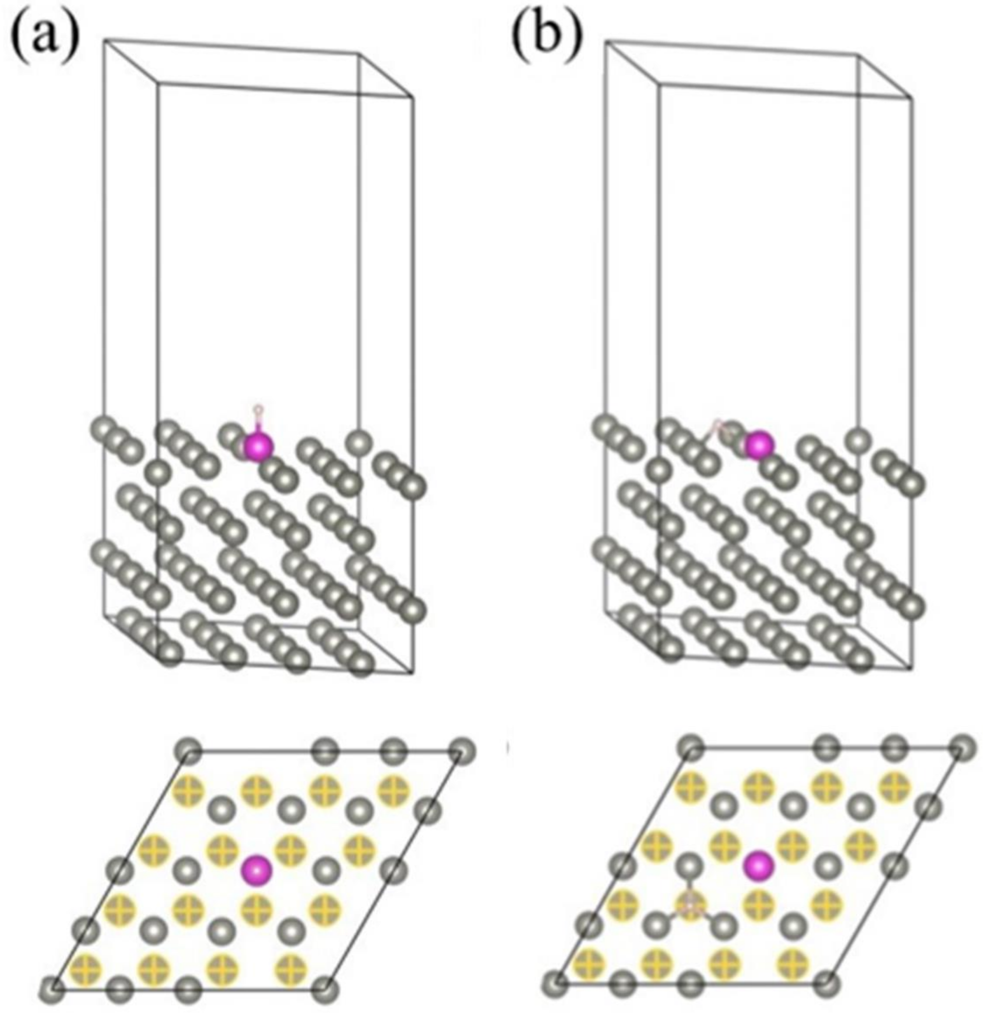

Figure S2. Adsorption sites of H atom on 4×4 ZnCd(001) supercell model. (a) Cd top. (b) Zn Hollow.

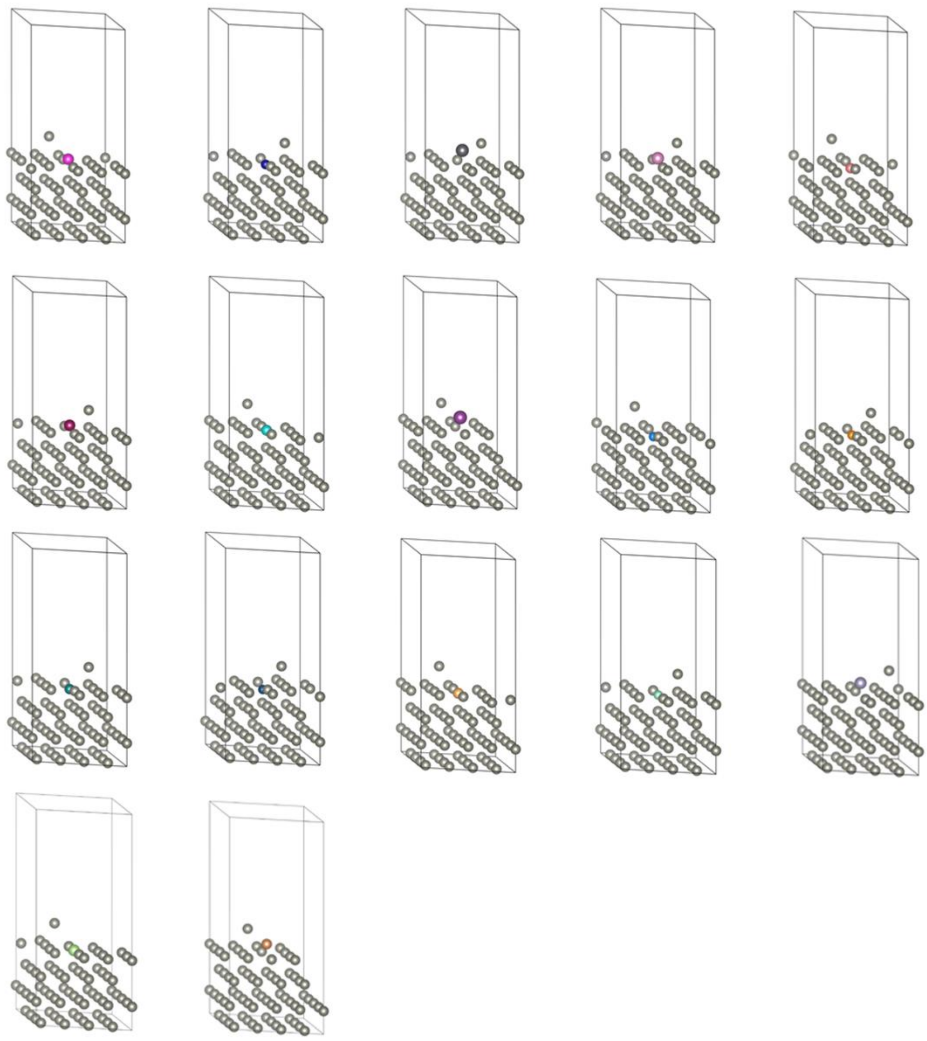

Figure S3. Zn atomic deposition model on ZnX(001) surface (doped element X In order of Cd, Cr, Pb, In, Pd, Hg, Ag, Bi, Co, Cu, Mn, Ni, Au, Ge, Sn, Ga, Sb)

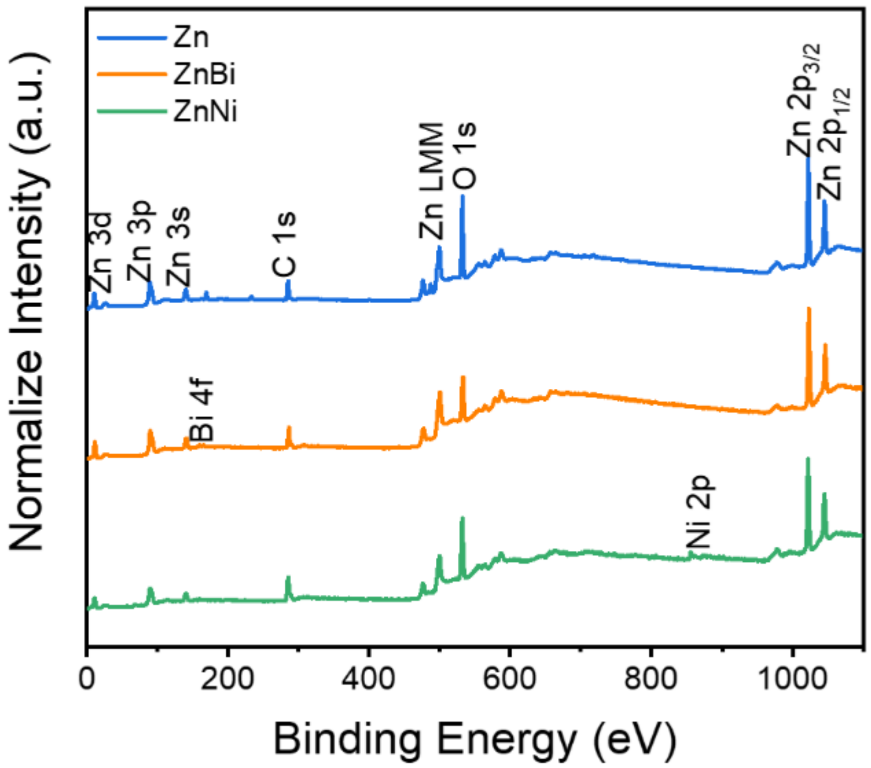

Figure S4. XPS survey spectra of pure Zn, ZnBi and ZnNi.

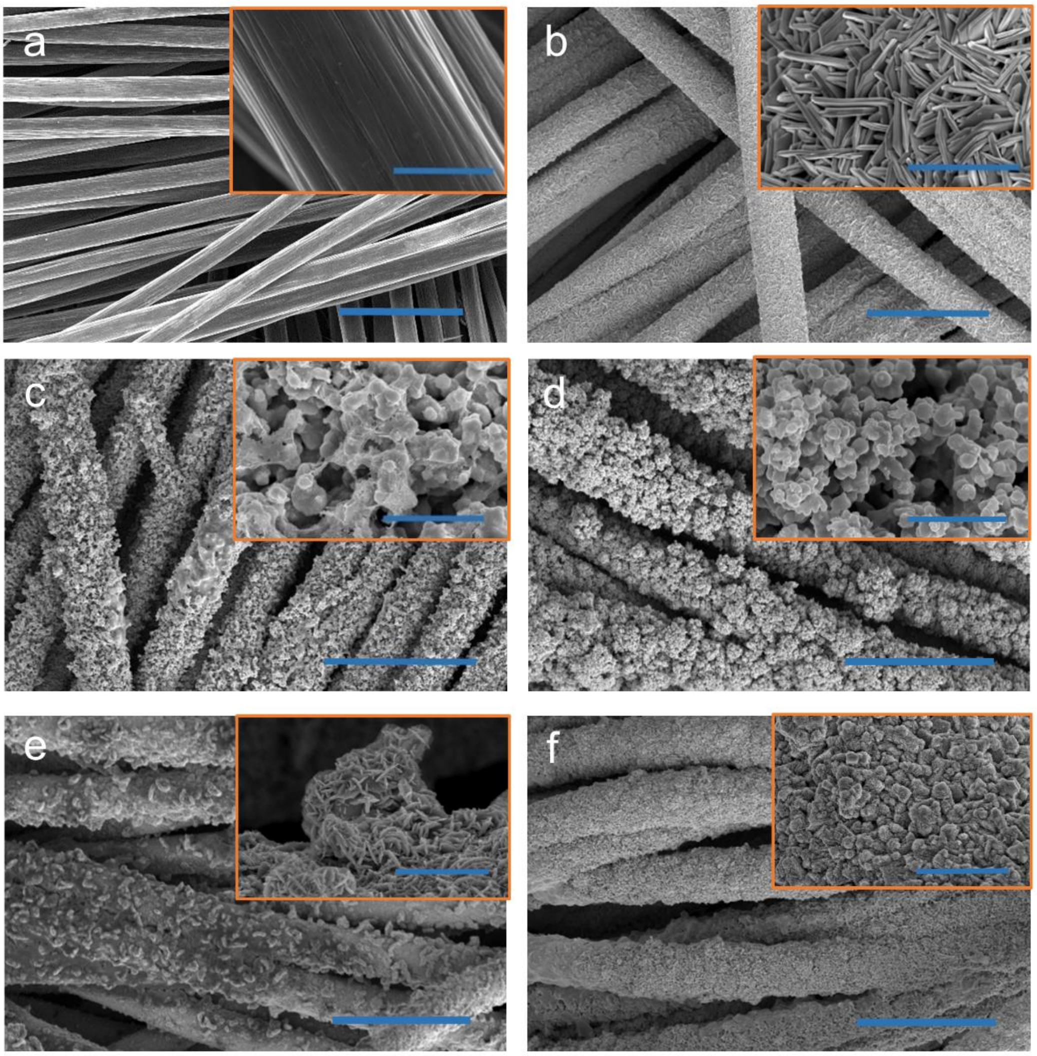

Figure S5. SEM images of (a) CNT, (b) pure Zn, (c) ZnBi, (d) ZnBi-2 (e) ZnNi and (f) ZnNi-2 samples. Scale bars, 50 μm (a–f) and 3 μm (insets of (a–f)).

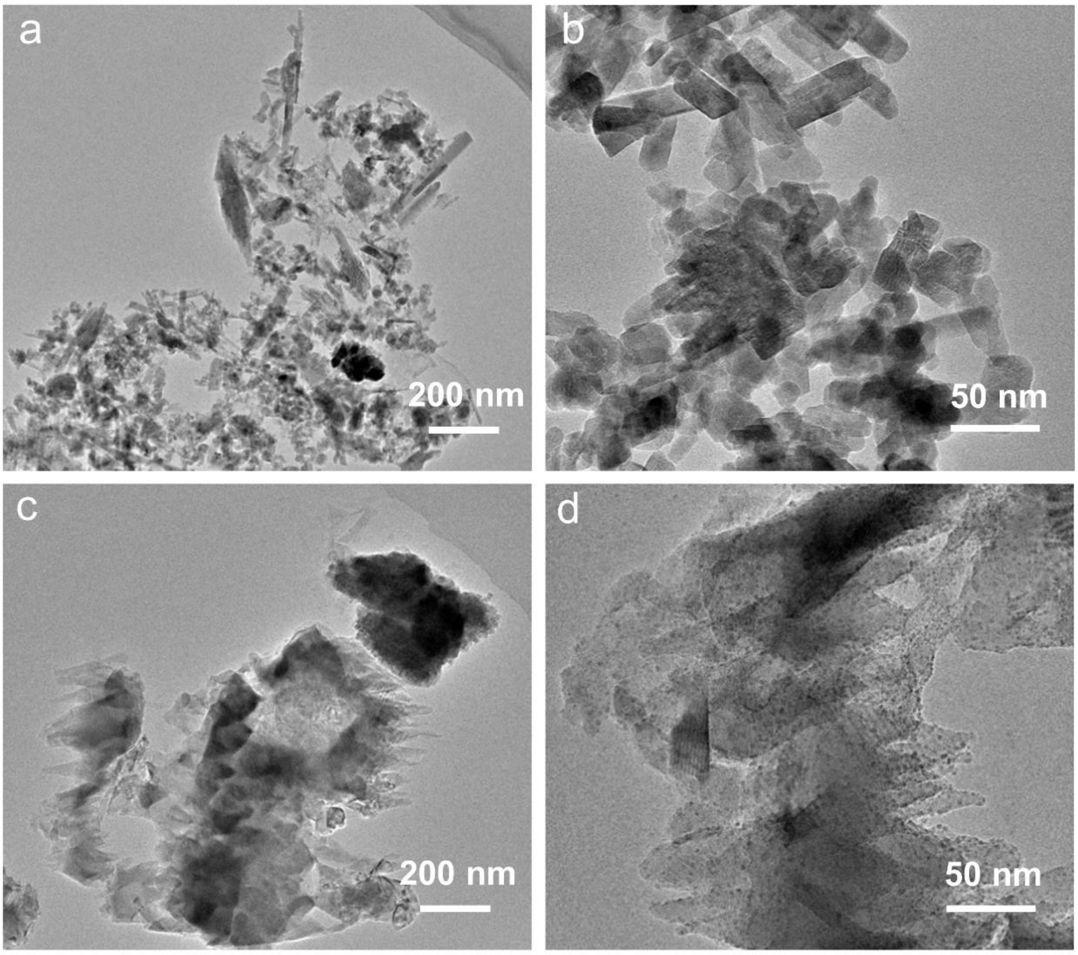

Figure S6. TEM images of (a,b) ZnBi and (c,d) ZnNi samples.

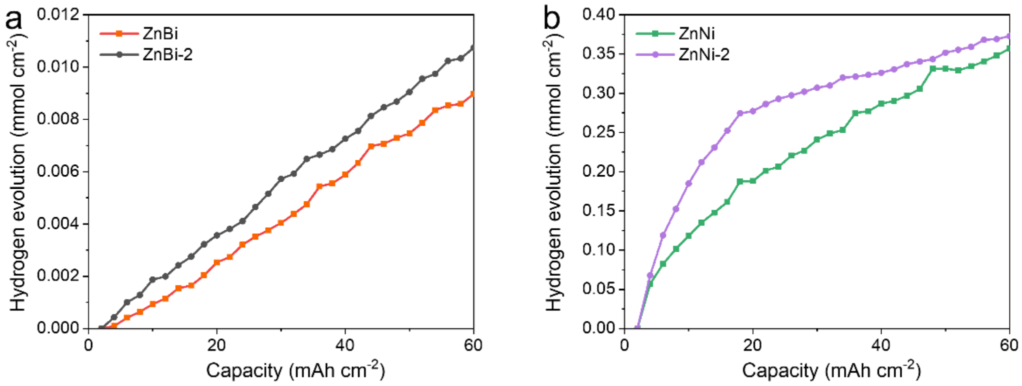

Figure S7. *In-situ* measurement of hydrogen evolution varying with the cumulative plating/stripping capacity of (a) ZnBi, ZnBi-2 and (b) ZnNi, ZnNi-2 symmetric cells at 2 mA cm^{-2} and 2 mAh cm^{-2}.

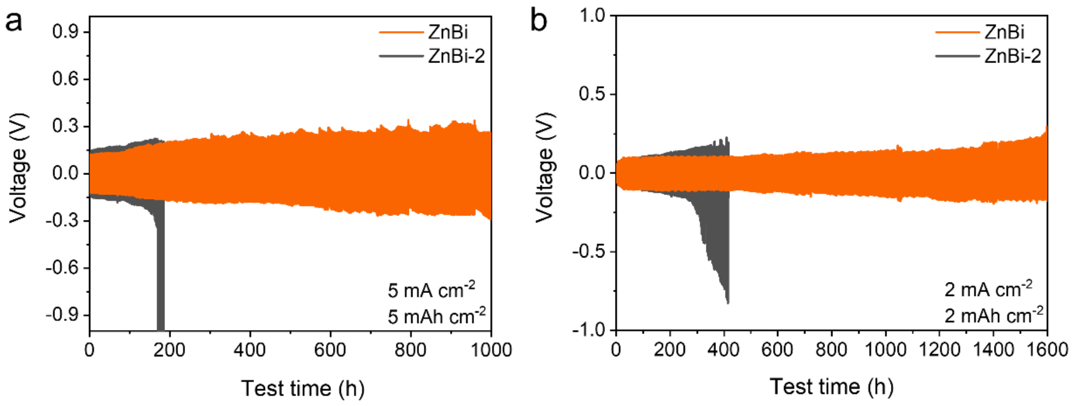

Figure S8. Comparison of the cycling performance of ZnBi and ZnBi-2 in symmetric cells at (a) 5 mA cm^{-2} for 5 mAh cm^{-2} and (b) 2 mA cm^{-2} for 2 mAh cm^{-2}.

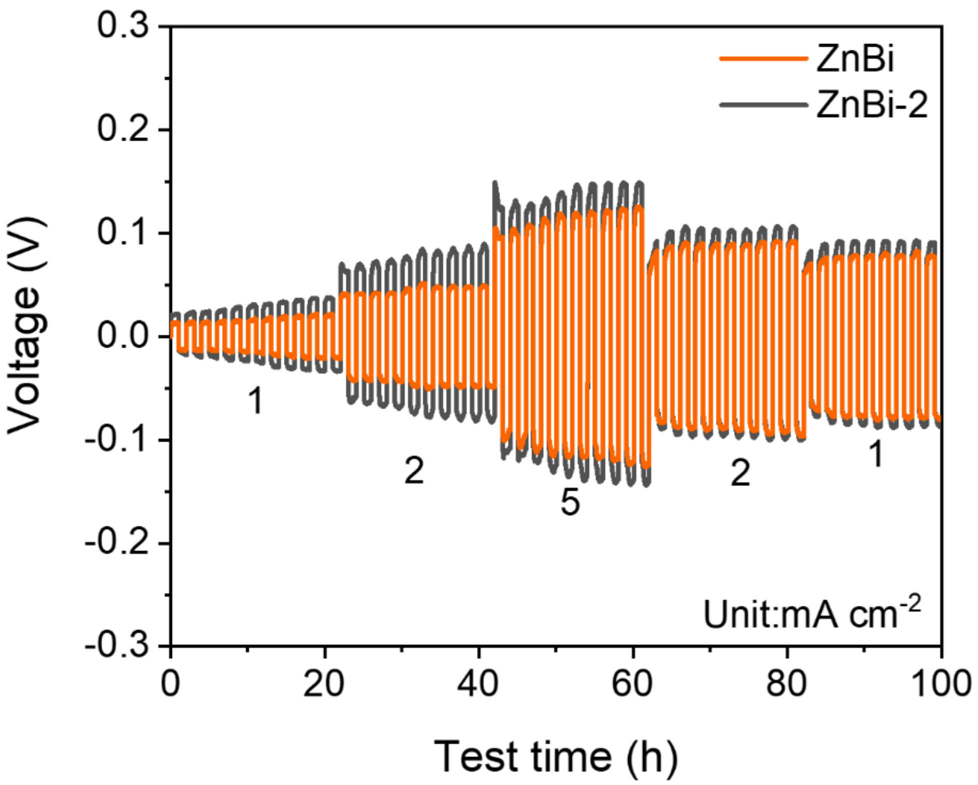

Figure S9. Rate performance of the symmetric cells based on ZnBi and ZnBi-2 anodes at current densities from 1 to 5 mA cm^{-2}.

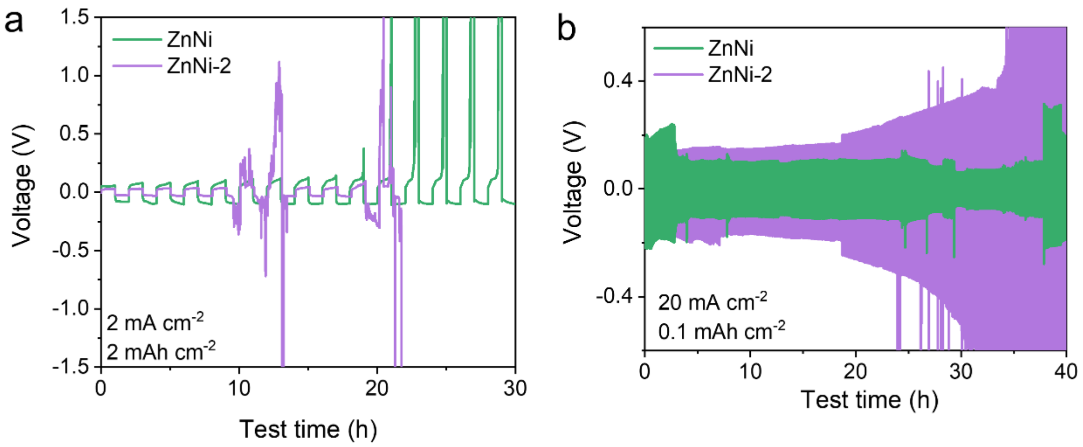

Figure S10. Comparison of the cycling performance of ZnNi and ZnNi-2 in symmetric cells at (a) 2 mA cm^{-2} for 2 mAh cm^{-2} and (b) 20 mA cm^{-2} for 0.1 mAh cm^{-2}.

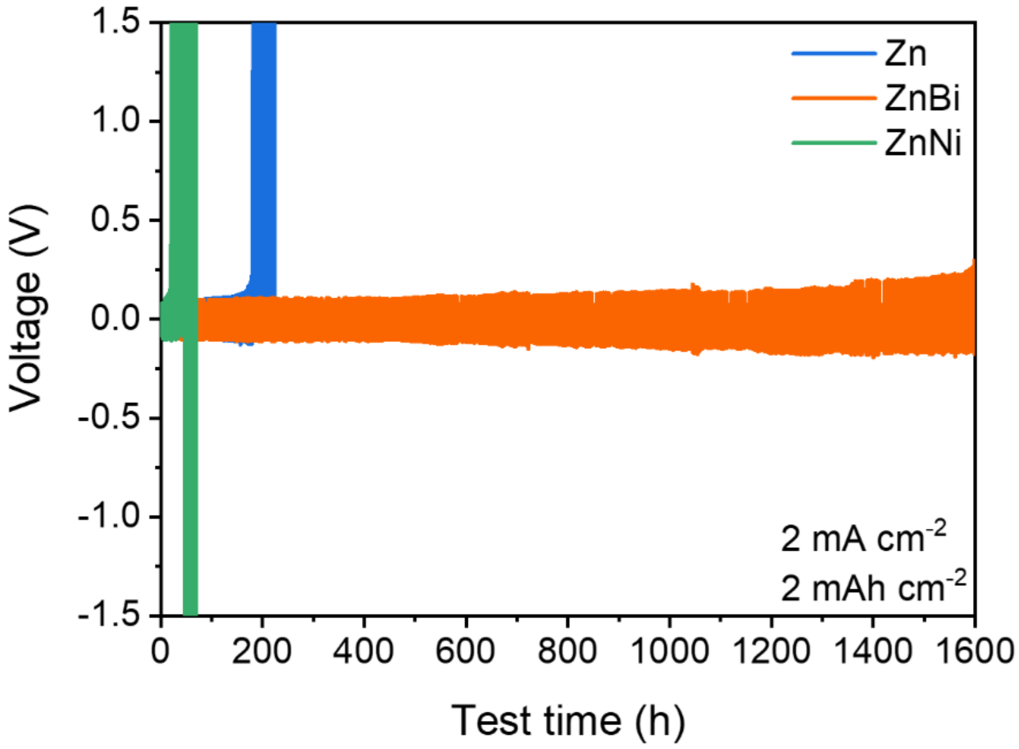

Figure S11. Comparison of the cycling performance of Zn, ZnBi and ZnNi in symmetric cells at 2 mA cm^{-2} for 2 mAh cm^{-2}.

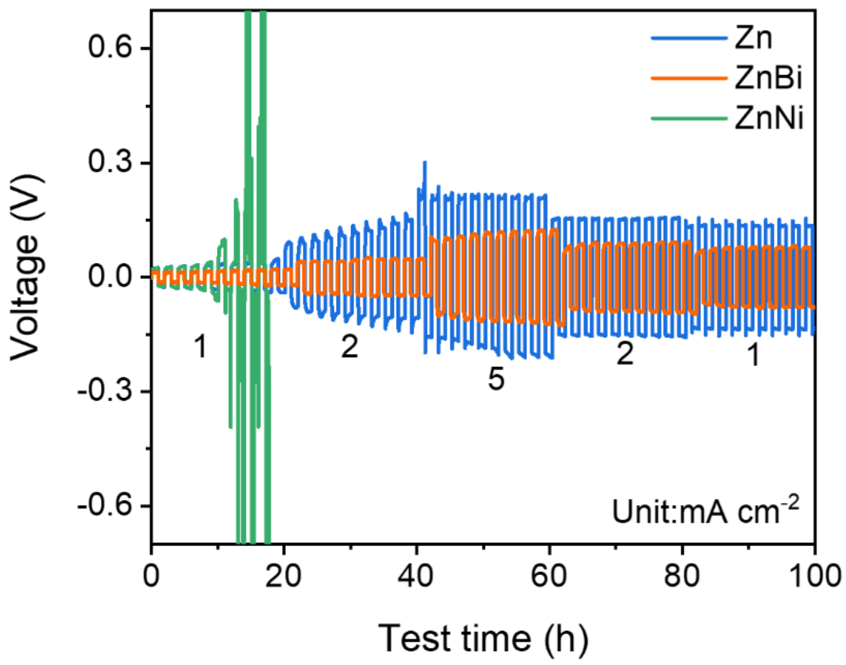

Figure S12. Rate performance of the symmetric cells based on Zn, ZnBi and ZnNi anodes at current densities from 1 to 5 mA cm^{-2}.

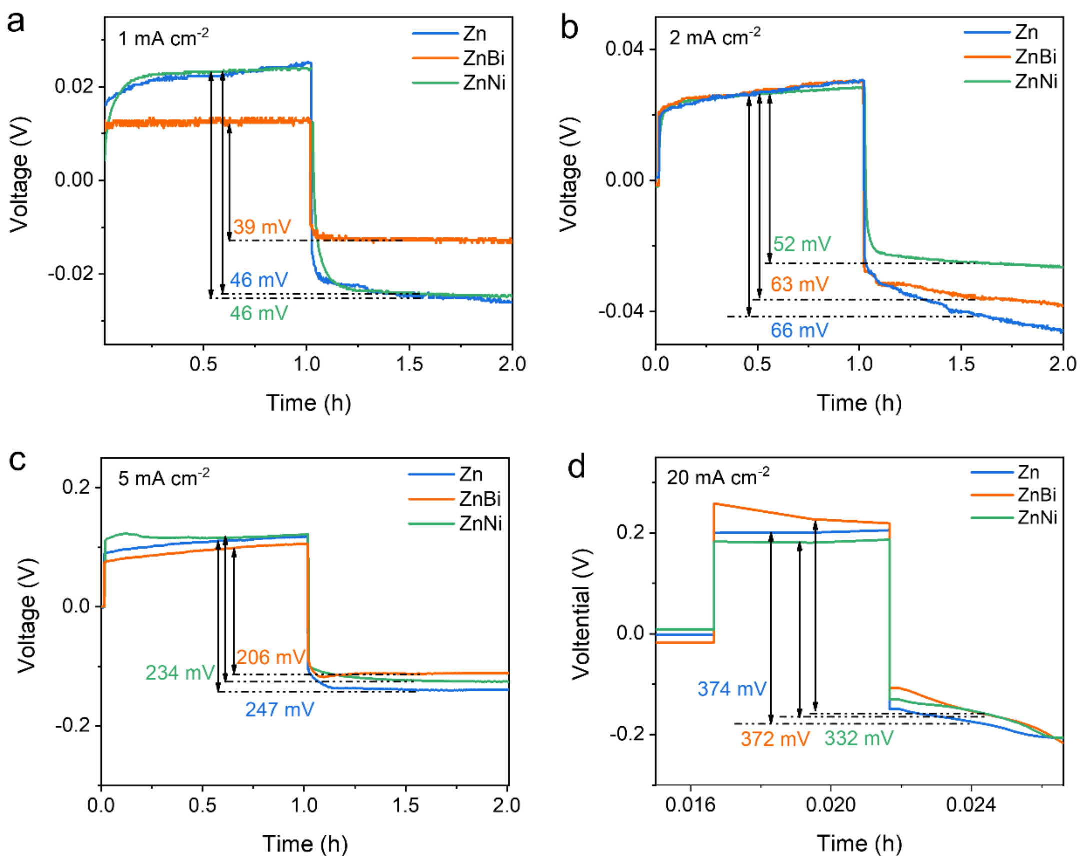

Figure S13. Overpotentials of the bare Zn, ZnBi and ZnNi electrodes at (a) 1 mA cm^{-2}, (b) 2 mA cm^{-2}, (c) 5 mA cm^{-2}, (d) 20 mA cm^{-2}.

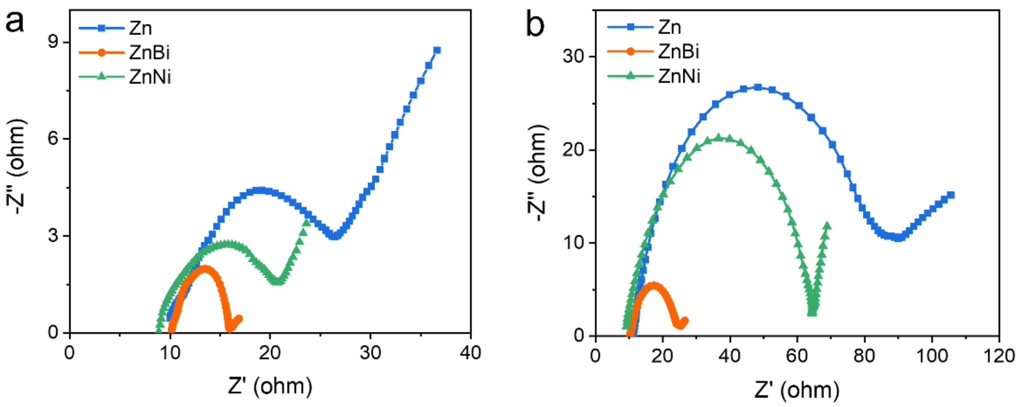

Figure S14. The electrochemical impedance spectroscopy of the symmetrical cells (a) before and (b) after 100 cycles at 2 mA cm^{-2} and 2 mAh cm^{-2} cutting-off capacity.

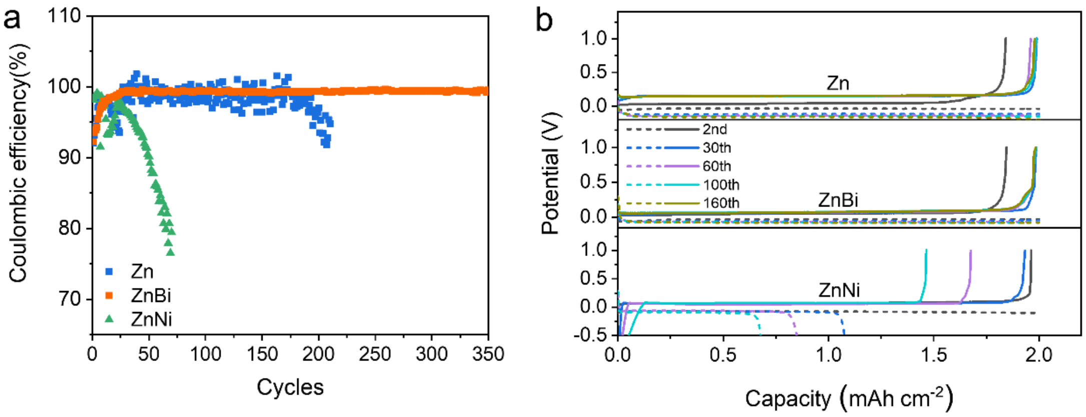

Figure S15. (a) Coulombic efficiencies of the Zn plating/stripping in Zn||Cu, ZnBi||Cu and ZnNi||Cu cells at 2 mA cm^{-2} and 2 mAh cm^{-2} cutting-off capacity. (b) Voltage profiles of Zn||Cu, ZnBi||Cu and ZnNi||Cu cells at the 2nd, 30th, 60th, 100th and 160th cycles.

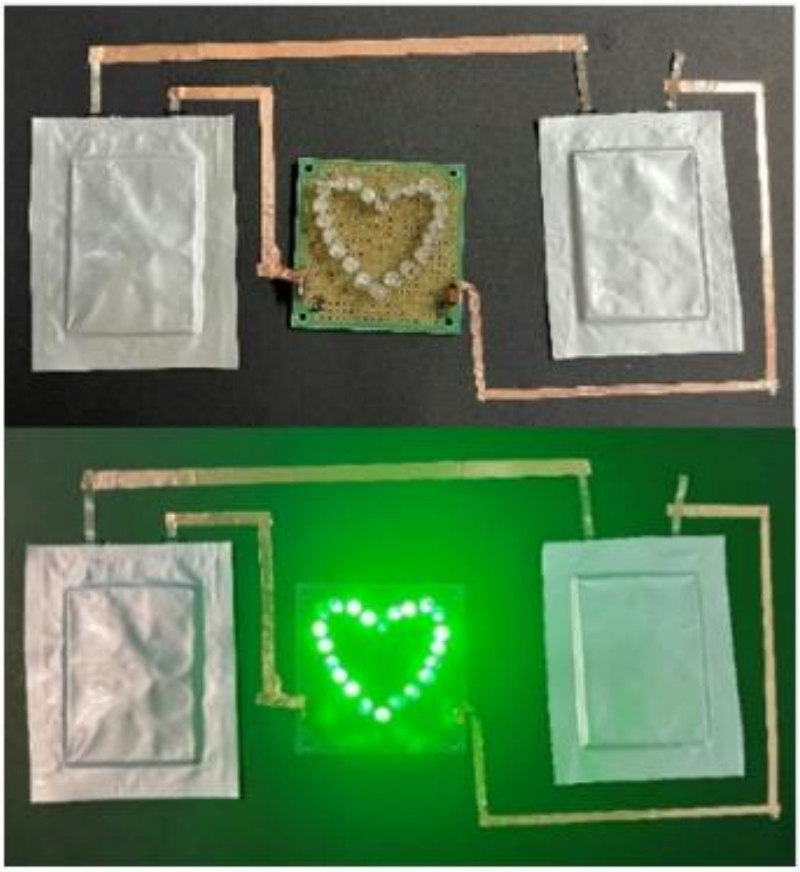

Figure S16. Digital photograph of two ZnBi||V_2O_5 cells powering heart-shaped lights.

References

[1] G. Kresse, J. Furthmüller, *Phys. Rev. B* **1996**, 54, 11169.

[2] P. E. Blöchl, *Phys. Rev. B* **1994**, 50, 17953.

[3] J. P. Perdew, K. Burke, M. Ernzerhof, *Phys. Rev. Lett.* **1996**, 77, 3865.

[4] L. Wang, W. Huang, W. Guo, Z. H. Guo, C. Chang, L. Gao, X. Pu, *Adv. Funct. Mater.* **2021**, 32, 2108533.

[5] Y. Jia, L. Z. Zhang, A. J. Du, G. P. Gao, J. Chen, X. C. Yan, C. L. Brown, X. D. Yao, *Adv. Mater.* **2016**, 28, 9532.

[6] P. Cao, X. Zhou, A. Wei, Q. Meng, H. Ye, W. Liu, J. Tang, J. Yang, *Adv. Funct. Mater.* **2021**, 31, 2100398.

[7] X. Xu, Y. Chen, D. Zheng, P. Ruan, Y. Cai, X. Dai, X. Niu, C. Pei, W. Shi, W. Liu, F. Wu, Z. Pan, H. Li, X. Cao, *Small* **2021**, 17, 2101901.

[8] S. Li, J. Fu, G. Miao, S. Wang, W. Zhao, Z. Wu, Y. Zhang, X. Yang, *Adv. Mater.* **2021**, 33, 2008424.

[9] J. Hao, B. Li, X. Li, X. Zeng, S. Zhang, F. Yang, S. Liu, D. Li, C. Wu, Z. Guo, *Adv. Mater.* **2020**, 32, 2003021.

[10] G. Liang, J. Zhu, B. Yan, Q. Li, A. Chen, Z. Chen, X. Wang, B. Xiong, J. Fan, J. Xu, C. Zhi, *Energy Environ. Sci.* **2022**, 15, 1086.

[11] X. Wang, J. Meng, X. Lin, Y. Yang, S. Zhou, Y. Wang, A. Pan, *Adv. Funct. Mater.* **2021**, 31, 2106114.

[12] X. Yang, C. Li, Z. Sun, S. Yang, Z. Shi, R. Huang, B. Liu, S. Li, Y. Wu, M. Wang, Y. Su, S. Dou, J. Sun, *Adv. Mater.* **2021**, 33, 2105951.

[13] W. Guo, Z. Cong, Z. Guo, C. Chang, X. Liang, Y. Liu, W. Hu, X. Pu, *Energy Storage Mater.* **2020**, 30, 104.

[14] W. Wang, G. Huang, Y. Wang, Z. Cao, L. Cavallo, M. N. Hedhili, H. N. Alshareef, *Adv. Energy Mater.* **2022**, 12, 2102797.

[15] J. Zheng, Z. Huang, Y. Zeng, W. Liu, B. Wei, Z. Qi, Z. Wang, C. Xia, H. Liang, *Nano Lett.* **2022**, 22, 1017.

[16] Y. Su, B. Chen, Y. Sun, Z. Xue, Y. Zou, D. Yang, L. Sun, X. Yang, C. Li, Y. Yang, X. Song, W. Guo, S. Dou, D. Chao, Z. Liu, J. Sun, *Adv. Mater.* **2023**, 35,

2301410.

[17] Q. Li, B. Yan, D. Wang, Q. Yang, Z. Huang, J. Fan, M. Dai, W. Chen, C. Zhi, *Small* **2022**, 18, 2201045.

[18] H. Li, C. Guo, T. Zhang, P. Xue, R. Zhao, W. Zhou, W. Li, A. Elzatahry, D. Zhao, D. Chao, *Nano Lett.* **2022**, 22, 4223.
